# Recomendaciones Sociedad Española de Investigación Ósea y Metabolismo Mineral (SEIOMM)-SEMERGEN-semFYC-SEMG para el manejo de los pacientes con osteoporosis/fractura por fragilidad en atención primaria

**DOI:** 10.1016/j.aprim.2026.103476

**Published:** 2026-03-04

**Authors:** Cristina Carbonell-Abella, Rafael Manuel Micó-Pérez, Francisco Vargas Negrín, José Carlos Bastida-Calvo, Mercè Giner, Pilar Aguado Acín

**Affiliations:** a Medicina Familiar y Comunitaria, Centro de Salud Vía Roma. Universitat de Barcelona, Barcelona, España; b Medicina Familiar y Comunitaria, Centro Fontanars dels Alforins. Departamento de salud Xátiva-Ontinyent. Grupo de trabajo de Aparato Locomotor, Sociedad Española de Médicos de Atención Primaria (SEMERGEN), Valencia, España; c Medicina de Familia, Centro de Salud Dr. Guigou. Grupo de Trabajo de Enfermedades Reumáticas y Musculoesqueléticas, Sociedad Española de Medicina de Familia y Comunitaria (semFYC), Tenerife, España; d Medicina Familiar y Comunitaria. Centro de Saúde Marín, Grupo Patología Osteoarticular/Osteoporosis Sociedad Española de Médicos Generales y de Familia (SEMG), Pontevedra, España; e Departamento de Citología e Histología Normal y Patológica, Facultad de Medicina de la Universidad de Sevilla. Sociedad Española de Investigación Ósea y del Metabolismo Mineral (SEIOMM), Sevilla, España; f Servicio de Reumatología. Hospital Universitario La Paz. Sociedad Española de Investigación Ósea y del Metabolismo Mineral (SEIOMM), Madrid, España

**Keywords:** Osteoporosis, Fractura por fragilidad, Atención primaria, Osteoporosis, Fragility fracture, Primary care

## Abstract

La osteoporosis (OP) se caracteriza por la disminución de la densidad mineral y el deterioro de la microarquitectura ósea, lo que incrementa la fragilidad y la susceptibilidad a fracturas. Las fracturas por fragilidad generan un elevado impacto clínico, social y económico, aumentando de forma significativa la morbimortalidad. Sin embargo, persiste una brecha relevante en su diagnóstico y tratamiento, especialmente en atención primaria.

Este documento presenta las primeras recomendaciones consensuadas por cuatro sociedades científicas (Sociedad Española de Médicos de Atención Primaria [SEMERGEN], Sociedad Española de Medicina de Familia y Comunitaria [semFYC], Sociedad Española de Médicos Generales y de Familia [SEMG] y Sociedad Española de Investigación Ósea y del Metabolismo Mineral [SEIOMM]), derivadas de un consenso Delphi y sustentadas en la evidencia científica más actual. Su objetivo es unificar criterios y mejorar la coordinación entre niveles asistenciales.

Las recomendaciones se estructuran en: identificación y evaluación del riesgo de fractura, tratamiento, seguimiento y criterios de derivación. Se propone el uso de herramientas de evaluación del riesgo de fractura a 10 años como *Fracture Risk Assessment Tool* (FRAX) y la densitometria ósea (DXA) para estratificar el riesgo y se establecen pautas claras para iniciar tratamiento según el perfil del paciente. Se enfatiza la importancia de la adherencia terapéutica, la reevaluación periódica y la participación del paciente en la toma de decisiones.

## Abstract (en)

Osteoporosis is characterized by decreased bone mineral density and deterioration of bone microarchitecture, leading to increased skeletal vulnerability and susceptibility to fracture. These fractures impose a substantial clinical, social, and economic burden, significantly elevating morbidity and mortality. However, a relevant gap persists in diagnosis and therapeutic management, particularly within primary care.

This document presents the first consensus recommendations developed by four scientific societies (SEIOMM, SEMERGEN, semFYC, and SEMG), derived from a Delphi process and supported by the most current scientific evidence. Its objective is to unify clinical criteria and improve coordination across healthcare levels.

The recommendations are structured around: identification and assessment of fracture risk, treatment, follow-up, and referral criteria. The use of 10-year fracture risk assessment tools such as FRAX (Fracture Risk Assessment Tool) and dual-energy X-ray absorptiometry (DXA) is proposed for risk stratification, and clear guidelines are established for initiating treatment based on the patient's profile. The importance of treatment adherence, periodic reassessment, and patient participation in decision-making is emphasized.

## Introducción

La osteoporosis (OP) es una enfermedad sistémica caracterizada por la reducción de la densidad mineral ósea (DMO) y el deterioro de la microarquitectura del tejido óseo, lo que incrementa la fragilidad ósea y la probabilidad de fracturas^1^. Estas fracturas, denominadas fracturas por fragilidad, tienen un impacto considerable sobre la calidad de vida, la autonomía funcional y la mortalidad, especialmente en la población de edad avanzada^2^.

En Europa, más de 32 millones de personas padecen OP, y en España afecta a casi tres millones de individuos, con una prevalencia estimada del 5,4%, siendo aproximadamente el 80% mujeres^3^. A pesar de su elevada carga clínica y socioeconómica, persiste una importante brecha en el diagnóstico y tratamiento de la OP, particularmente en atención primaria.

El estudio PREFRAOS, realizado en centros de salud españoles, puso de manifiesto que el 17,7% de las personas mayores de 70 años presentaba al menos una fractura por fragilidad, con una prevalencia tres veces superior en mujeres respecto a los hombres (24% frente a 8%). Además, solo el 65% de estos pacientes tenía un diagnóstico previo de OP, y el 57% no recibía tratamiento específico en el momento de la inclusión en el estudio, a pesar de tratarse de pacientes de alto riesgo^4^.

Otros estudios indican, que también en nuestro país, menos del 25% de los pacientes con fractura de cadera recibían tratamiento previo^5^, ^6^.

Aunque el manejo del evento agudo —la fractura— suele ser adecuado, con frecuencia no se identifica su origen como fractura por fragilidad ni se adoptan medidas dirigidas a prevenir nuevas fracturas, especialmente durante los dos años posteriores, periodo de máximo riesgo^7^. Esta situación genera un importante déficit en la prevención secundaria, estimándose que más del 45% de las personas con alto riesgo de nueva fractura no recibe ningún tipo de tratamiento antifractura^8^.

La variabilidad en la práctica clínica y la ausencia de protocolos homogéneos dificultan una prevención y un abordaje eficaces de la enfermedad. En este contexto, resulta imprescindible alcanzar consensos entre las distintas sociedades científicas que permitan establecer recomendaciones basadas en la evidencia, mejorar la coordinación entre niveles asistenciales y optimizar la atención a los pacientes con OP y fracturas por fragilidad^9^.

Las sociedades científicas desempeñan un papel esencial en este proceso. Cuatro entidades —Sociedad Española de Médicos de Atención Primaria [SEMERGEN], Sociedad Española de Medicina de Familia y Comunitaria [semFYC], Sociedad Española de Médicos Generales y de Familia [SEMG] y Sociedad Española de Investigación Ósea y del Metabolismo Mineral [SEIOMM]— han colaborado de forma activa en la elaboración del presente documento, aportando su experiencia clínica y científica. Esta colaboración ha sido clave para garantizar la calidad, el rigor metodológico y la validez de las recomendaciones, que se fundamentan en un consenso Delphi^10^ y en la mejor evidencia disponible.

Asimismo, estas sociedades han avalado el contenido del documento, subrayando su valor como herramienta para unificar criterios, reforzar la coordinación entre niveles asistenciales y promover una práctica clínica más homogénea y centrada en el paciente. Este esfuerzo conjunto refleja el compromiso de las sociedades científicas con la mejora de la atención primaria y la prevención de fracturas por fragilidad, abordando la heterogeneidad asistencial y fomentando el trabajo interdisciplinar.

## Metodología

### Recomendaciones para el manejo de los pacientes con osteoporosis

Estas recomendaciones surgen de un Consenso Delphi llevado a cabo por las distintas sociedades científicas de atención primaria (SEMERGEN, SemFyC y SEMG) en colaboración con la SEIOMM. El método Delphi, es una técnica de comunicación estructurada que permite recoger opiniones sobre un determinado tema complejo o controvertido para el que no se dispone de suficiente evidencia o su conocimiento es incompleto o incierto^11^, ^12^, ^13^. Además, gracias a este método se pueden explorar y unificar las opiniones de un grupo de expertos sin las dificultades e inconvenientes inherentes a los consensos presenciales, como los sesgos de influencia o interacción no confidencial.

El estudio fue llevado a cabo en varias fases: 1) creación de un comité científico de expertos con representantes de la SEMERGEN, SemFyC, SEMG y SEIOMM; 2) revisión de las principales guías nacionales e internacionales de referencia sobre el diagnóstico y tratamiento de la OP; 3) identificación de aquellos aspectos más controvertidos o con menor evidencia; 4) creación de un cuestionario Delphi con aseveraciones relativas a los aspectos identificados en el punto anterior; 5) dos rondas sucesivas en las que un panel de expertos mostró su grado de acuerdo con las aseveraciones propuestas; y 6) recopilación, análisis y discusión de los resultados. El panel de expertos fue elegido por el comité científico a partir de los socios de las diferentes sociedades médicas procurando disponer de una adecuada representación territorial de todas las Comunidades Autónomas de España. Las sociedades médicas participantes fueron: SEMERGEN, SemFyC, SEMG y SEIOMM. Esta última fue la impulsora del proyecto. Para analizar la opinión del panel de 77 expertos y el tipo de consenso alcanzado sobre cada aseveración planteada, se empleó la mediana y el intervalo intercuartílico de las puntuaciones obtenidas para cada aseveración. Se consideró que hubo consenso en cualquiera de ellas cuando dos tercios o más de los encuestados (≥ 66,7%) puntuaban dentro del rango de tres puntos (1-3 o 7-9) que contiene la mediana. El tipo de consenso alcanzado en cada aseveración se determinó por el valor de la mediana de la puntuación. Había consenso en el acuerdo si la mediana era ≥ 7 y había consenso en el desacuerdo si la mediana era ≤ 3. Se consideró que no hubo consenso cuando las puntuaciones de un tercio o más de los panelistas (≥ 33,3%) se situaban en el rango de 1 a 3 y otro tercio o más en el rango de 7 a 9. En la tabla 1 presentamos de forma resumida la tasa de consenso de cada ronda.

**Tabla 1:** Tasas de consensos alcanzados en el consenso Delphi^10^

Etapa	Ítems evaluados (n)	Consenso total n (%)	Sin consenso n (%)
Ronda 1	92	61 (66,3%)	31 (33,7%)
Ronda 2	31	16 (51,6%)	15 (48,4%)
Total final	92	77 (83,7%)	15 (16,3%)

Las recomendaciones se fundamentan en las aseveraciones consensuadas en dicho documento, así como en una exhaustiva revisión de la evidencia científica proveniente de las guías nacionales e internacionales más recientes^14^, ^15^, ^16^, ^17^, ^18^.

El propósito principal de estas recomendaciones es unificar los criterios para la identificación, evaluación, tratamiento y seguimiento de pacientes con OP, y definir la coordinación entre la atención primaria y el segundo nivel asistencial, en el contexto actual de heterogeneidad de recomendaciones de las distintas guías y actuaciones. Nuestro objetivo es proporcionar una herramienta útil para los profesionales sanitarios, especialmente para los médicos de atención primaria, en el manejo de la OP primaria y la prevención de fracturas en mujeres posmenopáusicas y varones mayores de 50 años.

Es importante señalar que estas directrices excluyen el manejo de la OP secundaria asociada a comorbilidades o fármacos que afectan la salud ósea. Asimismo, reconocemos las siguientes limitaciones: no abordan la prevención de caídas ni la identificación de la fragilidad, y la evidencia disponible para varones es limitada.

Para la formulación de estas recomendaciones, se ha considerado no solo el nivel de evidencia científica, sino también el balance riesgo-beneficio, los valores y preferencias de los pacientes, y la relación coste-beneficio.

La fortaleza de las recomendaciones se ha asignado según el nivel de evidencia científica en el que se basan:

• Se recomienda: Indica una recomendación fuerte (evidencia alta-moderada). La mayoría de los pacientes deberían recibir la intervención recomendada. En el texto se destaca las recomendaciones en marco gris.
• Se sugiere: Indica una recomendación condicional (evidencia moderada-baja-muy baja). Reconoce que diferentes opciones pueden ser apropiadas para cada paciente individual.

Siempre se debe fomentar que el paciente participe activamente en la toma de decisiones, alineando el manejo con sus valores y preferencias.

Estas recomendaciones y sugerencias se estructuraron a partir de los cuatro bloques principales establecidos en el documento de consenso original: identificación y evaluación, tratamiento, monitorización y seguimiento, criterios de derivación, punto de vista del paciente.

## Identificación y evaluación

### Concepto clave

Promover la concienciación de la OP y el conocimiento de su manejo en atención primaria. Identificar al paciente con OP y evaluar el riesgo de fractura por fragilidad del paciente.

### Recomendaciones

**Figura 1:**
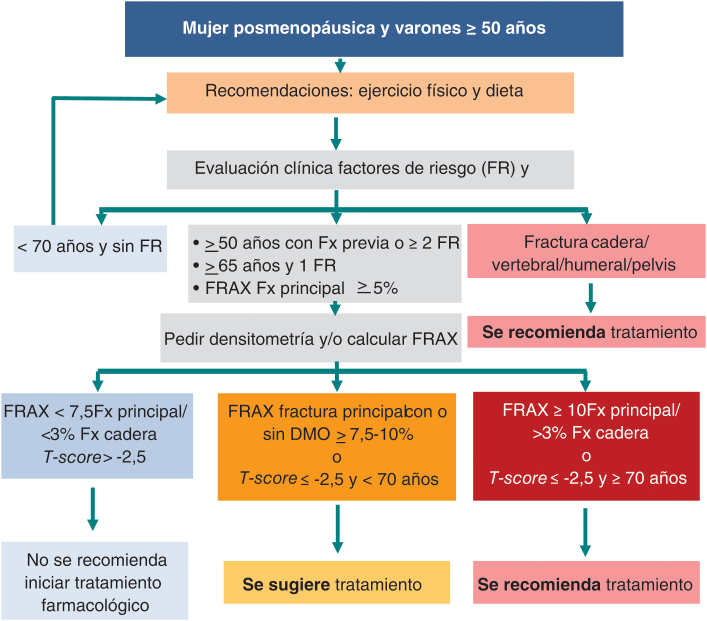
Algoritmo para la evaluación del riesgo de fractura e inicio del tratamiento. Adaptado de Morin et al.^17^.

1. Evaluar el riesgo de fractura por fragilidad teniendo en cuenta la edad, los factores de riesgo clínicos de fractura y la medición de la DMO y estratificar el riesgo de fractura de los pacientes: muy alto, alto o moderado (fig. 1) (tabla 2).

**Tabla 2:** Estratificación del riesgo de fractura

**Muy alto riesgo**
-Pacientes con dos o más fracturas vertebrales -Una fractura vertebral severa (Grado 3 de la clasificación de Genant^19^; reducción altura cuerpo vertebral > 40%) (figura suplementaria 1). -Fractura vertebral o de cadera asociada a *T-score* < −2,5 -DMO muy baja (*T-score* < −3,5)
**Alto riesgo**
-Fractura por fragilidad de localización no incluida en el grupo de muy alto riesgo -*T-score* < −2,5 y edad ≥ 70 años (*T-score* < -2,5 DE en personas de > 70 años) -FRAX® para fractura de cadera ≥ 3% -FRAX fractura principal con/sin DMO >7,5%-10% -Pacientes con osteopenia que presenten otros factores de riesgo mayores
**Moderado riesgo**
-Pacientes con *T-score*< −2,0 en cadera (osteopenia) y/o *T-score*< −2,5 (osteoporosis) -en columna lumbar, con edad < 70 años y sin fracturas
2. Valorar los factores de riesgo clínicos fuertemente asociados a la fractura (tabla 3).

**Tabla 3:** Factores de riesgo clínicos valorables en la enfermedad osteoporótica^14^. RR: riesgo relativo

Mayores (RR x ≥ 2)	Menores (RR 1-2 veces)
Edad > 65 años Bajo peso, índice de masa corporal < 20 kg/m^2^ Antecedente personal de fractura por fragilidad Antecedente familiar de fractura de cadera Corticoterapia (> 5 mg/día x 3 meses o más) Caídas frecuentes (≥ 2 al año)	Tabaquismo activo Consumo de alcohol (> 3 unidades/día) Menopausia precoz (< 45 años) Artritis reumatoide Diabetes Hipertiroidismo no corregido Otras enfermedades (Crohn, hepatopatía crónica, nefropatía, etc.) Otros fármacos (inhibidores de la aromatasa, inhibidores selectivos de la recaptación de serotonina, anticonvulsivantes, bloqueo androgénico, inhibidores de la bomba de protones, antirretrovirales)
3. Evaluar el riesgo de caídas por su relevancia en el riesgo de fractura.
4. Realizar un estudio analítico básico para descartar causas secundarias.
5. Realizar una radiografía lateral de columna dorsal y lumbar cuando haya sospecha de fractura vertebral (por dolor, cifosis o pérdida de estatura), exposición a glucocorticoides, mujeres de edad > 65 años y con osteopenia o cuando iniciamos un tratamiento antiosteoporótico.
6. Valorar la prevención secundaria de fractura siempre que exista una fractura por fragilidad sobre todo cuando esa fractura sea reciente (dos últimos años), ya que se considera como un elemento multiplicador para la estratificación del riesgo de fractura.

Se sugiere utilizar la herramienta FRAX con/sin el valor obtenido en la DMO para la evaluación del riesgo de fractura^20^, sobre todo en la prevención primaria de fractura. Se sugiere clasificar a los pacientes como de alto riesgo de fractura (umbral de tratamiento) cuando el riesgo cuantificado por FRAX sin DMO para fractura principal sea > 10% (con DMO > 7,5%), y/o el riesgo para fractura de cadera sea > 3%^21^.

FRAX es útil para evaluar el riesgo de fractura de cadera tanto en hombres como en mujeres. La herramienta FRAX no debe sustituir el juicio clínico del médico que es quien valora todos los factores de riesgo y la situación clínica del paciente.

Se sugiere la medición de la DMO mediante densitometria ósea (DXA) (absorciometría dual de rayos X) en las siguientes condiciones:

a) Pacientes > 50 años con fractura previa por fragilidad o > 2 factores de riesgo clínico.
b) Pacientes > 65 años con 1 factor de riesgo clínico.
c) Pacientes con resultado de FRAX para fractura principal > 5%

## Tratamiento

### Concepto clave

Es fundamental determinar a quien y cuando tratar, así como la elección del tratamiento y establecer su secuencia y duración.

a) Medidas no farmacológicas:

### Recomendaciones

A las mujeres posmenopáusicas y a los hombres de edad ≥ 50 años con OP o en riesgo de fractura por fragilidad se les recomienda mantener una dieta equilibrada con un aporte de proteínas de 1-1,5 g/kg y realizar ejercicio físico regular, incluyendo ejercicios de entrenamiento funcional y del equilibrio, adaptados a las necesidades y capacidades de cada paciente, y evitar el tabaquismo y el consumo excesivo de alcohol. Se recomiendan consejos para la prevención de caídas.

Se sugiere realizar una ingesta diaria de calcio entre 1.000 y 1.200 mg, preferiblemente mediante la dieta o con suplementos si con esta no se alcanzase. Una toma diaria de vitamina D entre 800-1200 UI/día en pacientes con OP o con riesgo de fractura o si se ha detectado insuficiencia de vitamina D o factores de riesgo de esta. Dado que es difícil alcanzar este nivel de ingesta o si las fuentes naturales son insuficientes se recomienda suplementar. Las personas confinadas en casa o que viven en residencias tienen más probabilidades de necesitar suplementos de calcio y vitamina D para alcanzar los niveles recomendados. Las concentraciones séricas de 25-hidroxivitamina D (25OHD) deseables para asegurar una buena salud ósea se sitúan por encima de 30 ng/mL.

Los pacientes tratados con fármacos para la OP (antirresortivos o anabólicos) deben recibir un aporte adecuado de calcio y de vitamina D.

b) Inicio del tratamiento farmacológico

### Recomendaciones

Se recomienda iniciar tratamiento farmacológico para reducir el riesgo de fractura osteoporótica en las siguientes situaciones (fig. 1):

1. Pacientes con fractura por fragilidad vertebral, cadera, húmero o pelvis. Tras una fractura por fragilidad, el diagnóstico de OP e inicio de tratamiento debe hacerse tan pronto como sea posible.
2. Pacientes con una puntuación de T-score ≤ -2,5 en columna vertebral, cuello femoral o cadera total y con una edad ≥ 70 años.
3. Pacientes que presentan un FRAX para fractura de cadera ≥ 3%.
4. Pacientes con osteopenia (particularmente si la puntuación T-score es ≤ −2,0) que presentan, además, alto riesgo clínico de fractura (2 factores de riesgo clínico fuertemente asociados a fractura, FRAX principal ≥ 10% sin DMO o ≥ 7,5% con DMO y FRAX de cadera ≥ 3%).
5. Pacientes en tratamiento crónico con glucocorticoides orales, inhibidores aromatasa y fármacos de privación androgénica siguiendo las guías «*ad hoc*» para estas situaciones de valoración del riesgo de fractura y de tratamiento.

**Figura 2:**
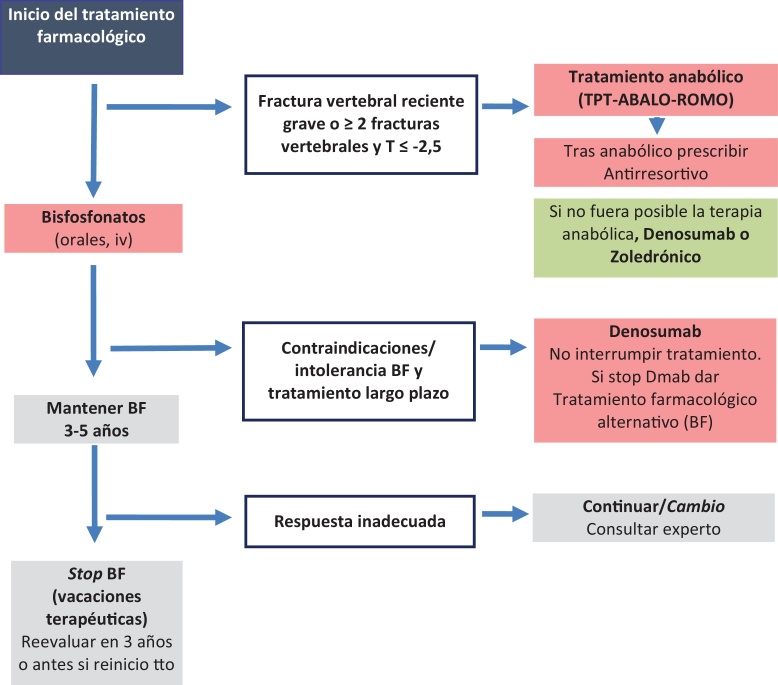
Algoritmo de tratamiento farmacológico, duración y secuencia del tratamiento. Adaptado de Morin et al.^17^. BF: bifosfonato; TPT: teriparatida; ABALO: abaloparatida; ROMO: romosozumab; IV: intravenoso.

Se sugiere consensuar con el paciente e iniciar tratamiento farmacológico para reducir el riesgo de fractura osteoporótica en las siguientes situaciones:

i) Pacientes que presentan un FRAX para fractura principal ≥10% sin DMO o ≥7,5% con DMO.
ii) Pacientes con una puntuación *T-score* ≤ -2,5 en columna, cuello femoral o cadera total y con una edad ≤ 70 años.
iii) Pacientes ≤ 70 años con fractura de radio distal (sin OP densitométrica) y en aquellos que presentan deformidades vertebrales grado 1 en la radiología simple de columna (reducción altura de cuerpo vertebral del 20%-25%).

c) Elección del fármaco:

La elección del fármaco debe de realizarse de manera individualizada teniendo en cuenta diferentes factores (fig. 2):

a) Eficacia y seguridad de los fármacos.
b) Estratificación del riesgo de fractura (edad, factores de riesgo clínicos y valor de la DMO).
c) La presencia de fracturas, número, localización y la cronología de estas.
d) Características individuales del paciente (comorbilidad, polimedicación, adherencia al tratamiento y preferencias).
e) La relación coste-efectividad.

### Recomendaciones

1. En pacientes con riesgo alto o moderado de fractura se recomienda iniciar tratamiento con bisfosfonatos orales en ausencia de intolerancia digestiva o contraindicación^14^, ^15^, ^16^, ^17^, ^18^.
2. En pacientes con riesgo alto o moderado de fractura con intolerancia digestiva, comorbilidad, polimedicados, con baja adherencia o > 75 años, se recomienda tratamiento parenteral con ácido zoledrónico o denosumab. En la elección entre ambos, deben considerarse que el ácido zoledrónico está contraindicado si el filtrado glomerular es inferior a 35mL/min, mientras que el riesgo de posible disminución de masa ósea rápida y aparición de fracturas vertebrales múltiples tras la suspensión de denosumab limita su indicación en pacientes con baja adherencia o edad joven. La administración de ácido zoledrónico se realiza en el hospital^14^, ^15^, ^16^, ^17^, ^18^.
3. En pacientes con muy alto riesgo de fractura (una fractura vertebral grave reciente o ≥ 2 fractura vertebral y una puntuación T-score ≤ -3,0) se recomienda considerar la terapia secuencial con inicio de un anabólico^22^, ^23^ y en aquellos pacientes que no sea viable, el ácido zoledrónico y el denosumab son alternativas eficaces^14^, ^15^, ^16^, ^17^, ^18^.

### Adherencia y persistencia al tratamiento

**Figura 3:**
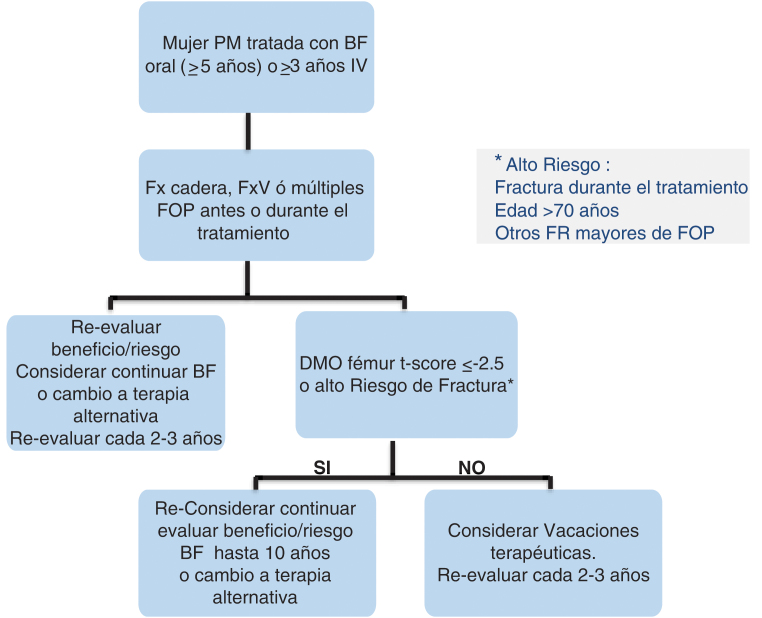
Algoritmo de vacaciones terapéuticas. Adaptado de Adler et al.^24^. PM: posmenopaúsica; BF: bifosfonatos; FV: fractura vertebral; FOP: fractura osteoporótica principal o mayor.

• Se recomienda: Realizar una evaluación periódica de la adherencia y persistencia al tratamiento farmacológico, identificando barreras y facilitando estrategias para mejorarla (por ejemplo, simplificación de la pauta, educación, programas de soporte al paciente).

d) Vacaciones terapéuticas:

La aparición, aunque poco frecuente, de efectos adversos serios como la osteonecrosis mandibular y las fracturas atípicas de fémur se ha relacionado con el uso prolongado de tratamientos antirresortivos, en particular bifosfonatos (BF) y denosumab. Este hecho unido a la conocida permanencia prolongada en el esqueleto de los BF planteó hace ya casi una década la necesidad de reevaluar periódicamente la indicación terapéutica e indicar si procede vacaciones terapéuticas^24^ (fig. 3).

### Recomendaciones

1. En los pacientes que reciben tratamiento con BF, valorar la suspensión temporal del fármaco tras tres años de tratamiento intravenoso (IV) o cinco años de tratamiento oral. Esta decisión debe basarse en una reevaluación integral del riesgo individual de fractura y considerando el fármaco empleado. No implica necesariamente la interrupción para todos los pacientes, sino una evaluación clínica que permita decidir si hay que continuar o hacer una pausa en la terapia^25^.

De forma general, se aconseja mantener el tratamiento hasta seis años (BF IV) o hasta 10 años (BF orales) en pacientes con factores de alto riesgo como:

• antecedentes de fractura por fragilidad o durante el tratamiento
• con *T-score* en cadera ≤ -2,5 y edad ≥ 70-75 años
• uso prolongado de glucocorticoides

En pacientes con riesgo bajo o moderado, puede indicarse una interrupción del tratamiento (pausa terapéutica), con un seguimiento clínico (por si aparecen nuevos factores de riesgo o fracturas incidentes) y densitométrico cada dos a tres años para valorar la necesidad de reintroducir el tratamiento.

En el caso del denosumab, el enfoque es distinto, ya que su efecto antirresortivo es reversible tras la suspensión. Se han documentado casos de fracturas vertebrales múltiples después de interrumpir su administración sin una transición adecuada a otro tratamiento antirresortivo. El tratamiento con denosumab no debe interrumpirse temporalmente. Si esto fuera preciso por algún motivo, debe hacerse con una estrategia de transición a otro fármaco, un bisfosfonato, alendronato o zoledronato (siendo este último preferible si el tratamiento con denosumab se prolongó durante más de dos a tres años), para evitar el posible riesgo de disminución de masa ósea rápida y aparición de fracturas vertebrales múltiples^15^, ^26^, ^27^.

## Monitorización y seguimiento

Se recomienda para hacer el seguimiento de pacientes con OP:

1. El uso de densitometría ósea mediante DXA en intervalos adecuados. En pacientes en tratamiento con antirresortivos medir la DMO con intervalos largos (tres a cinco años).
2. Realizar evaluación individualizada para asegurar eficacia y seguridad del tratamiento.
3. Realizar de forma compartida la evaluación, el tratamiento y el seguimiento entre las Unidades de Coordinación de Fracturas/expertos en metabolismo óseo y AP. Las fracturas de cadera, por su morbimortalidad, requieren una especial atención.

## Criterios de derivación

### Concepto clave

Es una prioridad establecer y difundir protocolos claros de coordinación y derivación entre atención primaria y atención hospitalaria, incluyendo el uso de e-consultas cuando sea posible, para asegurar la continuidad asistencial y el manejo integral del paciente con OP. La interconsulta sin pacientes, entre profesionales, puede ser una herramienta útil para favorecer esta mejor atención.

Se recomienda, establecer criterios de coordinación y derivación consensuados entre atención primaria y atención hospitalaria. Se consideran como criterios de derivación:

a. La sospecha de OP secundaria.
b. La OP juvenil.
c. La respuesta inadecuada al tratamiento (no debida a la falta de cumplimiento) con progresión significativa de la pérdida de DMO o nuevas fracturas.
d. La presencia de efectos secundarios/contraindicaciones al tratamiento que dificultan el manejo terapéutico.
e. La presencia de patologías concomitantes que hacen que los pacientes sean especialmente complejos para su manejo terapéutico.
f. Cuando se considere oportuno tratamiento con BF intravenoso (zoledronato)

## Punto de vista del paciente

### Concepto clave

Se considera relevante incorporar en el manejo de la OP en atención primaria de manera sistemática la perspectiva del paciente, asegurando que este reciba información clara y comprensible sobre su enfermedad y tratamiento, confirmando su comprensión, implicándole en la toma de decisiones compartidas y valorando periódicamente su adherencia al tratamiento.

También se considera el fomentar la participación en programas de atención al paciente que refuercen la educación, el autocuidado y el seguimiento.

Es esencial implementar estrategias de comunicación efectiva que evalúen sus conocimientos previos sobre la OP, garanticen la comprensión de la información (p. ej., *Teach-back*^28^) y fomenten la toma de decisiones compartidas alineada con sus valores, preferencias y objetivos (calidad de vida, autonomía funcional, miedo a fracturas o efectos adversos). Las ayudas para la decisión (PDA) validadas en OP —disponibles en plataformas como el OP *Decision Aid* del IOF o el iFraP *intervention*— mejoran el conocimiento del paciente, reducen la indecisión terapéutica y facilitan discusiones centradas en preferencias como la vía/frecuencia de administración y el perfil de seguridad, incrementando la satisfacción^29^, ^30^, ^31^, ^32^, ^33^.

Para optimizar la adherencia y persistencia (objetivo > 70%, clave para reducir refracturas en 20-30%), se recomienda una evaluación periódica mediante escalas validadas (MARS-5 o BMQ), programas estructurados de *Fracture Liaison Services* (FLS) con recordatorios y soporte multidisciplinar, y el uso de apps educativas como Bone Matters o recursos de la SEIOMM/IOF, que han demostrado mejorar la persistencia hasta un 50% en prevención secundaria^34^, ^35^.

Estos enfoques, integrados en atención primaria, fortalecen la alianza terapéutica y reducen el infratratamiento.

### Recomendaciones

1. Garantizar que la información general y de autocuidados y del tratamiento que se le da al paciente, este la comprenda y entienda para mejorar los resultados del tratamiento.
2. Toma de decisiones compartidas con el paciente.
3. Valorar siempre de forma periódica el cumplimiento y persistencia del tratamiento por parte del paciente.

No fue objetivo del presente trabajo definir indicadores mínimos de calidad asistencial, ya que estos no fueron consensuados previamente por el panel de expertos al no estar incorporados en el cuestionario. Sin embargo, aunque existe esta limitación, creemos de gran relevancia formular indicadores de aspecto científico-técnico que se derivarían de la implementación de las recomendaciones y que deberían ser validados en futures acciones de consenso.

Los indicadores determinados según cada bloque de recomendaciones son:

#### 1. Identificación y evaluación

1a. Criterio: Los pacientes deben ser evaluados y estratificados según su riesgo de fractura. Indicador: n° de pacientes evaluados /n° total de pacientes atendidos en consulta por OP (x100).

Estándar: 100%

1b. Criterio: La solicitud de la densitometría se realizará después de valorar el riesgo de fractura

Indicador: n° de densitometrías pedidas que cumplen criterios de solicitud /n° total de densitometrías pedidas (x100).

Estándar: >80%

#### 2. Tratamiento y adecuación

2a. Criterio: Los pacientes con fractura de cadera por fragilidad, deben recibir un tratamiento osteoporótico al alta hospitaria, de acuerdo con los umbrales de tratamiento propuestos.

Indicador: n° de pacientes con fractura de cadera por fragilidad con tratamiento para la OP al alta hospitalaria/n° total de pacientes con fractura de cadera por fragilidad dados de alta (x100).

Estándar: 100%

#### 3. Seguimiento y coordinación

3a. Criterio: Los pacientes con fractura de cadera por fragilidad deben tener al menos una visita médica antes de los 12 meses tras el alta hospitalaria.

Indicador: n° de pacientes con fractura de cadera por fragilidad con revisión durante el año posterior al alta médica / n° total de pacientes con fractura de cadera por fragilidad dados de alta en el año previo (x100).

Estándar: 90%

3b. Criterio: los pacientes con criterios de complejidad (posible OP secundaria, mala respuesta, necesidad de BF IV, comorbilidad relevante) deben ser derivados según protocolo a unidades especializadas (unidades de metabolismo óseo/FLS) o mediante e-consulta. Indicador: n° de pacientes con criterios de complejidad derivados presencial o por e-consulta/n° total de pacientes con criterios de complejidad atendidos en consulta (x100).

Estándard: 90%

#### 4. Perspectiva del paciente

4a. Criterio: los pacientes con OP atendidos en consulta que inician tratamiento farmacológico deben recibir una evaluación periódica anual de la adherencia mediante escalas validadas.

Indicador: n° de pacientes que inician tratamiento farmacológico con evaluación al año de la adherencia/n° total de pacientes con OP que inician tratamiento farmacológico (x100)

Estándard:75%

4b. Criterio: Los pacientes con fractura de cadera por fragilidad deben tener al menos una intervención educativa antes de los 12 meses tras el alta hospitalaria. Indicador: n° de pacientes con fractura de cadera por fragilidad con intervención educativa durante el año tras el alta hospitalaria / n° total de pacientes con fractura de cadera por fragilidad dados de alta en el año previo (x100).

Estándar: 70%

**Lo conocido**

• La OP y las fracturas por fragilidad son frecuentes en la población atendida en atención primaria y se asocian a un elevado impacto clínico, funcional y en mortalidad.
• Tras una fractura por fragilidad, el riesgo de sufrir nuevas fracturas es especialmente alto durante los dos primeros años, lo que hace fundamental la prevención secundaria.
• Existe una brecha asistencial significativa en el diagnóstico y tratamiento de la OP, incluso en pacientes que ya han presentado una fractura.

**¿Qué aporta este estudio?**

• Presenta recomendaciones consensuadas entre cuatro sociedades científicas para el manejo de la OP y las fracturas por fragilidad en atención primaria.
• Refuerza la identificación sistemática y la evaluación estructurada del riesgo de fractura mediante herramientas como FRAX y la densitometría ósea.
• Propone criterios claros para la estratificación del riesgo, el inicio del tratamiento, el seguimiento y la coordinación entre atención primaria y otros niveles asistenciales, con el objetivo de reducir nuevas fracturas.

## Financiación

No se recibió financiación para la preparación de este manuscrito.

## Contribución de los autores

Todos los autores han contribuido por igual.

## Uso de IA

El uso de IA se ha limitado a la corrección gramatical del texto en inglés

## Consideraciones éticas y consentimiento informado

No proceden.

## Conflicto de intereses

Los autores declaran no tener ningún conflicto de intereses.

## Anexo A Material suplementario

Figura Suplementaria 1. Medición de las deformidades vertebrales, clasificación de Genant^19^
