# Supplementary figures and images for: SEIOMM-SEMERGEN-semFYC-SEMG recommendations for the management of patients with osteoporosis/fragility fracture in primary care
Source: Aten Primaria. 2026 Mar 4;58(5):103476. [Article in Spanish] doi: 10.1016/j.aprim.2026.103476 (PMC12971984; doi:10.1016/j.aprim.2026.103476)

Suplementaria Fig. I:

**
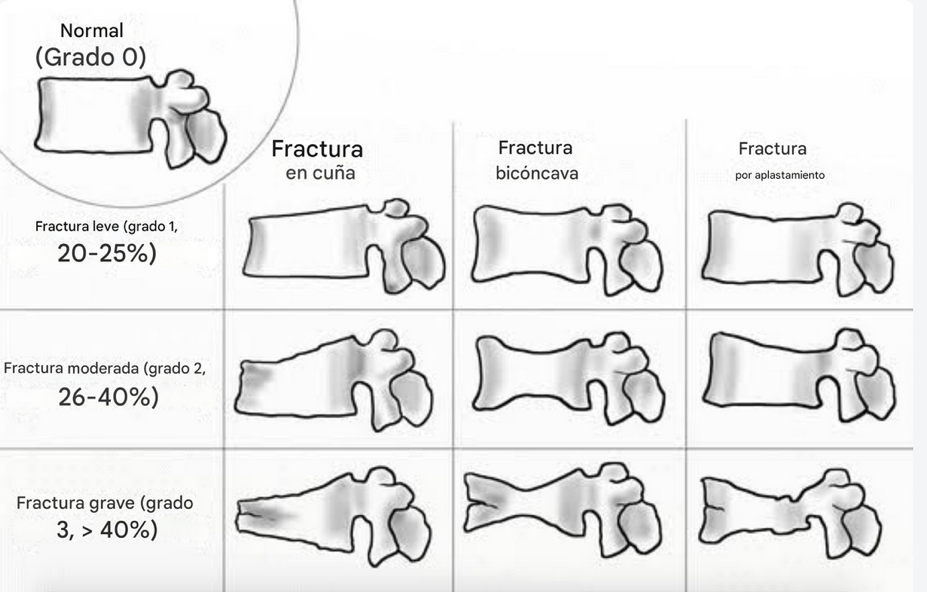
**

Supplement: Supplementary file 1 [file mmc1.doc]
